# Super-resolution visualization of chromatin loop folding in human lymphoblastoid cells using interferometric photoactivated localization microscopy

**DOI:** 10.1038/s41598-022-12568-9

**Published:** 2022-05-20

**Authors:** Zofia Parteka-Tojek, Jacqueline Jufen Zhu, Byoungkoo Lee, Karolina Jodkowska, Ping Wang, Jesse Aaron, Teng-Leong Chew, Krzysztof Banecki, Dariusz Plewczynski, Yijun Ruan

**Affiliations:** 1 The Jackson Laboratory for Genomic Medicine, 10 Discovery Drive, Farmington, CT 06030 USA; 2 Centre of New Technologies, University of Warsaw, S. Banacha 2c, 02-097 Warsaw, Poland; 3 Faculty of Mathematics and Information Science, Warsaw University of Technology, Warsaw, Poland; 4 Department of Genetics and Genome Sciences, University of Connecticut Health Center, 400 Farmington Avenue, Farmington, CT 06030 USA; 5 Centre for Advanced Materials and Technologies, Warsaw University of Technology, Poleczki 19, 02-822 Warsaw, Poland; 6 Advanced Imaging Center, Janelia Research Campus, Howard Hughes Medical Institute, 19700 Helix Drive, Ashburn, VA 20147 USA

**Keywords:** Epigenomics, Gene regulation, Genome, Genomics, Sequencing, Biophysics, Computational biology and bioinformatics, Software

## Abstract

The three-dimensional (3D) genome structure plays a fundamental role in gene regulation and cellular functions. Recent studies in 3D genomics inferred the very basic functional chromatin folding structures known as chromatin loops, the long-range chromatin interactions that are mediated by protein factors and dynamically extruded by cohesin. We combined the use of FISH staining of a very short (33 kb) chromatin fragment, interferometric photoactivated localization microscopy (iPALM), and traveling salesman problem-based heuristic loop reconstruction algorithm from an image of the one of the strongest CTCF-mediated chromatin loops in human lymphoblastoid cells. In total, we have generated thirteen good quality images of the target chromatin region with 2–22 nm oligo probe localization precision. We visualized the shape of the single chromatin loops with unprecedented genomic resolution which allowed us to study the structural heterogeneity of chromatin looping. We were able to compare the physical distance maps from all reconstructed image-driven computational models with contact frequencies observed by ChIA-PET and Hi-C genomic-driven methods to examine the concordance between single cell imaging and population based genomic data.

## Introduction

In eukaryotic cells, DNA is packed at multi-scale levels to allow meters-long linear genomic sequences to be condensed in a micrometers-sized nucleus. At the finest scale, 146 base pairs (bp) of DNA bound by histone proteins form nucleosomes^1,2^ that are connected by dozens of bp of linker DNA, appearing as a “beads on a string” structure^3–8^. The 10 nm “beads on a string” DNA fiber is then folded into higher-order chromatin structures for further chromatin compaction. Although the 30 nm chromatin fiber was observed in vitro and suggested to be the next organizational level of the 10 nm fiber, it is still debatable whether it exists in vivo^9–14^. In order to unravel the higher-order chromatin structure in vivo, technologies combining biochemistry and high-throughput sequencing (chromatin conformation capture (3C) methods) such as Hi-C^15^ and ChIA-PET^16^ have been developed. They allow to capture chromatin conformation by detecting DNA loci that are in close spatial proximity even though they may be located far away in linear genomic distance. With chromatin interaction information, DNA loops and higher-order chromatin structures such as megabase-sized topologically associating domains (TADs)^17^ and CTCF-mediated chromatin contact domains (CCDs)^18^ were detected. At the larger scale, domains group into chromatin compartments, which subsequently fold into chromatin territories that occupy specific regions of the nucleus^19^. Microscopy based techniques together with 3C methods revealed the existence of two types of compartments: A and B^20^, that correspond to transcriptionally active, early replicating euchromatin and transcriptionally repressed, late replicating heterochromatin respectively^21^. Heterochromatin is located towards the nuclear periphery and at nucleoli, whereas active genes and enhancers rich euchromatin is localized in the nuclear interior^20^. Interestingly the borders of replication timing domains showed nearly one-to-one correlation with TAD borders^22^. Moreover, alteration of TADs was demonstrated to affect local enhancer-promoter interactions leading to changes in gene expression^23^, in some cases to oncogene activation^24,25^ and was also linked to human disease^26^. These findings further emphasize the tight connection between genome structure and function.

It is proposed that TADs are formed in the loop extrusion (LE) process^27^. According to the LE model, an extrusion factor such as ring-shaped protein complex cohesin is loaded on a particular chromatin locus and a tiny loop is formed. Subsequently, cohesin traverses along the chromatin, increasing in the loop size, until it reaches an insulator such as the CTCF bound region^28^. LE model is supported by the findings that depletion of cohesin or CTCF led to the disappearance of loop domains^29,30^ and increased loading of cohesin on chromatin induced strengthening of loops at TAD borders^31^. Importantly, a different mechanism is thought to govern formation of A and B compartments^32^. As shown by Hi-C experiments as well as live-cell and super-resolution 3D microscopy approach, chromatin compartmentalization is maintained even after cohesin depletion^30,33^. Compartmentalization is tightly linked to the chromatin epigenetic landscape and is proposed to be driven by phase separation^34^. Strikingly, polymer simulation analysis revealed that LE competes with compartment segregation^35^ in line with previous experimental observation that downregulation of chromatin bound cohesin led to enhanced compartmentalization^36,37^. These findings indicate that the interplay between both processes shapes chromatin structure in the nucleus and suggest that loop domains and compartments are not hierarchical structures.

However, the visualization of higher-order chromatin structure in situ remains challenging mainly due to the difficulties in specific DNA labeling and limitations of microscopy resolution^38^. Recently, tremendous progress has been made in chromatin imaging by the introduction of novel DNA staining methods such as oligopaints^39,40^ combined with advanced microscopic technologies such as electron microscopy (EM), photoactivated localization microscopy (PALM) and stochastic optical reconstruction microscopy (STORM)^41,42^. In general, the genomic features of TADs or TAD-like domains visualized by microscopes are consistent with those inferred by Hi-C or ChIA-PET^43–47^. Notably, Bintu et al. demonstrated the TAD or TAD-like structures with clear boundaries in single cells using microscopy approaches^45^. At the finer scale chromatin ultra-structures such as nucleosome fibers were observed in situ in different cell stages and cell types, suggesting CTCF-based looping structures^14,48–50^ as the building blocks of chromatin higher order organization. However, due to the lack of specificity of DNA staining, the structure of chromatin loop in a specific chromatin region has not been yet addressed. The combination of FISH probes with different spectral signatures and super-resolution microscopy was used to observe gene domains in 3D in early 2000s^51^. Cremer and colleagues were pioneers in the application of localization microscopy (SPDM) for the nanostructural analysis of specific FISH-labelled chromatin domains which was used for the analysis of the spatial distribution of specific DNA sequences in intact human cell nuclei with 20 nm mean localization precision^52^. Recently, a novel method called optical reconstruction of chromatin architecture (ORCA) has been developed to image specific genomic regions with nanoscale accuracy and genomic resolution of up to two kilobases, allowing to visualize chromatin folding within small-scale (100–700 kb) regions and interactions between regulatory elements^53^ in Drosophila.

In our study, we aimed to visualize the shortest up to date human DNA region of a 13 kb chromatin loop (and 10 kb flanking region on each side of the loop) inferred by both ChIA-PET and Hi-C in human lymphoblastoid cell line using interferometric photoactivated localization microscopy (iPALM). The advantage of our approach is that we can observe single oligo probes attached to DNA fiber. In ORCA the goal was to observe a group of oligo probes staining a given region. Even with 1 kb spaced oligonucleotides, we would get 33 data points from our region. This would not be better than the resolution achieved by Hi-C population data. We resolved the detailed spatial conformation of the target 13 kb chromatin loop with 4–22 nm microscopic localization precision in X, Y, Z and between 300 and 120 bp genomic resolution, providing the first insight into how a chromatin loop detected by Hi-C and ChIA-PET experiments folds in single cells and how the imaging visualization can be directly correlated with population-averaged genomic data. In our work we show the high concordance between those two types of experimental methods targeted to represent the spatial structure of chromatin at the nanometer resolution.

## Results

### The chromatin landscape and experimental design on the target loop region

**Figure 1:**
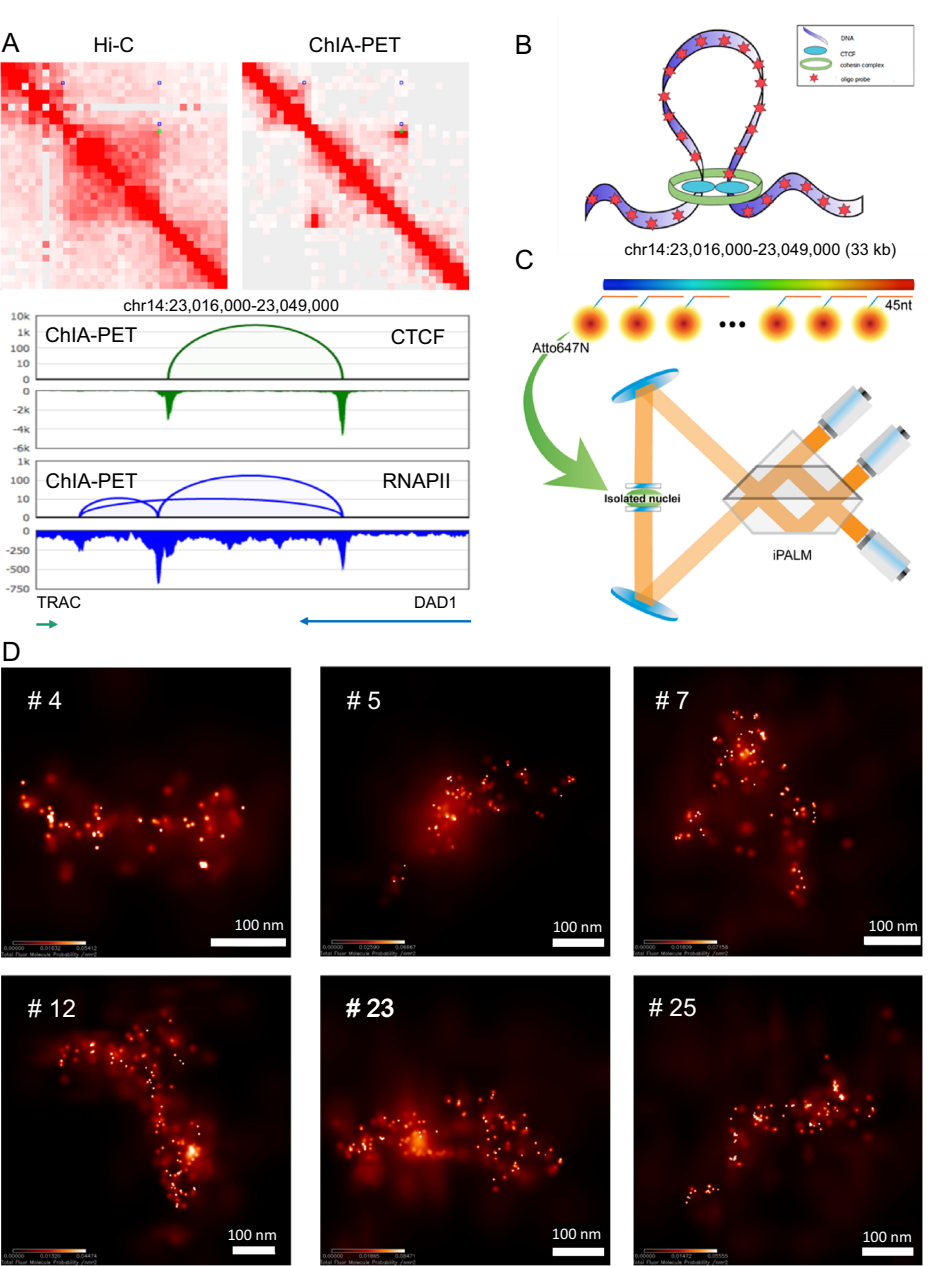
iPALM imaging of a target chromatin loop region. (**A**) GM12878 Hi-C^54^ (top left) and CTCF and RNAPII merged ChIA-PET^18^ (top right) contact map for the target region chromosome 14: 23.016–23.049 Mb (hg19, 1 Kb resolution with balanced normalization), as well as ChIA-PET genome browser view for the same region (bottom). ChIA-PET loops (green for CTCF and blue for RNAPII) are also presented on top of the upper diagonal area of the contact maps. In the browser view, CTCF loops and peaks (green), RNAPII loops and peaks (blue), and genes localized in the target region are presented. (**B**) Schematic of the target loop region inferred by ChIA-PET data with attached oligopaint probe. (**C**) Schematic of the iPALM method. The probe set contains 336 DNA oligos tagged with Atto647N designed to stain along the target chromatin region (**D**) Representative images of the target region from 6 different cells obtained by iPALM.

Hi-C^54^ and ChIA-PET^18^ data detected chromatin loops at various genomic loci and length scales. As a target chromatin region, we selected a 13 kb long, high frequency (Table S1, Table S2, Figure S1) loop mediated by both CTCF and RNA polymerase II (RNAPII) proteins in GM12878 cells together with flanking regions of 10 kb on each side (Fig. 1A). The loop resides on chromosome 14 within chromatin compartment A1, which is gene-rich, transcriptionally active and early replicating region, occupied by active epigenetic marks such as H3K27ac, H3K36me3, H3K4me1 (Figure S2, Rao et al., 2014). It is located near the T-cell receptor alpha (TCRA) locus, where V(D)J recombination takes place during T-lymphocyte development, which has been extensively studied in mouse T-cells^55–57^. A CTCF and cohesin-binding site is found to reside at the intergenic region between TCRA and the neighboring housekeeping gene DAD1. It is suggested that this site functions as an insulator, as the depletion of cohesin led to increased transcription of DAD1^58^. Interaction between a TCRA enhancer and DAD1 was shown to occur in both mouse T- and B-lymphocytes, although in the latter it was slightly weaker^57,58^. The conservation of TCRA locus between mouse and human^59^ implies a chromatin organization in human lymphocytes similar to the one observed in a mouse. As expected, in our GM12878 ChIA-PET data, one anchor of the loop overlaps with the TCRA enhancer and the other one is situated in the DAD1 gene body (Fig. 1A), which is like the observation in mouse T- cells, suggesting the insulation function of the target loop in human cells.

To keep the integrity of the target loop in consideration of staining efficiency, we extended the staining region by 10 kb on both sides of the loop. The resulting 33 kb chromatin region is presented in both the Hi-C^54^ heat map and ChIA-PET^18^ browser view (Fig. 1A). The surrounding epigenomic landscape of this region is shown in Figure S1. To enable fluorescent labeling on the target region (Fig. 1B), we designed Oligopaint probe^39^ for DNA fluorescence in situ hybridization (FISH). The probe is composed of a pool of 336 different DNA oligonucleotides (oligos) tagged with Atto647N dye. Each oligo consists of 45 nucleotides (nt) complementary to the target DNA sequence (Fig. 1C), resulting in a staining density of 11.57 oligos/kb along the loop region, and 6.7 and 11.5 oligos/kb at the two flanking sites. The Oligopaint probe was designed for the target with high specificity and low background noise by avoiding DNA repeats. Based on staining efficiency achieved by our experiments (Figure S22B) we can estimate that we managed to get the genomic resolution between 300 and 120 bp. To calculate the genomic resolution, we divided the length of the studied region (~ 33 kb) by the number of oligoprobes identified in images used for modelling (110–275 probes).

### iPALM imaging on the target chromatin loop

**Figure 2:**
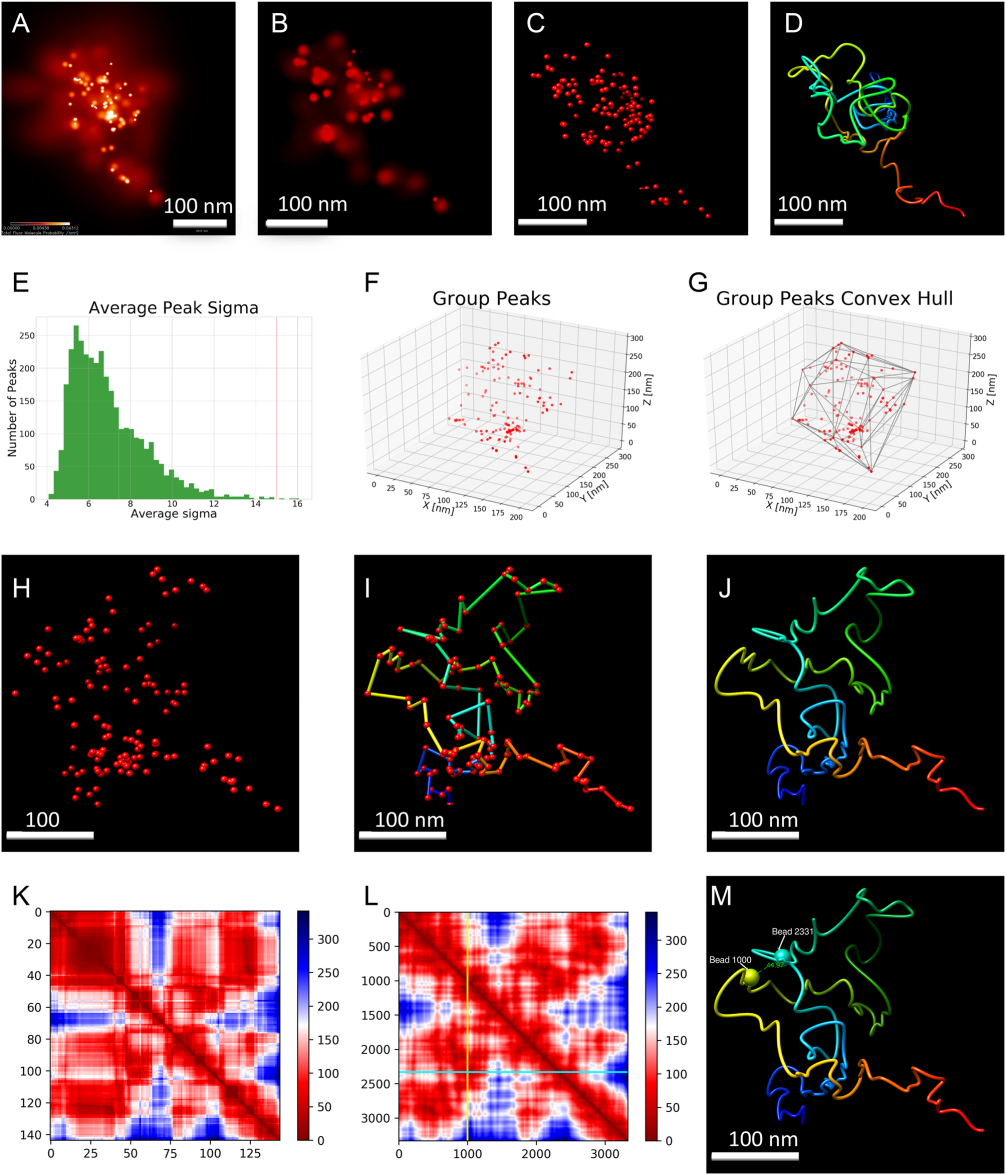
Processing and modeling pipeline for iPALM images based on image #17. (**A**) 2D image of the target chromatin region generated by PeakSelector. Color bar represents the probability of molecule presence per nm^2^ with white and dark red colors representing the high and low probability respectively. (**B**) 3D image of the target chromatin region (**C**) Group peaks visualized as points in 3D space. (**D**) 3D polymer model reconstructed from group peaks. The blue color represents the beginning of the model structure, red color represents the end. (**E**) Average sigma for each peak in the image. (**F**) 3D position plot of group peaks. (**G**) The convex hull of group peaks. (**H**–**J**) Modeling pipeline used to reconstruct chromatin loop in image # 17 (**H**) Group peaks in 3D space (**I**) Model generated by connecting group peaks using travelling salesman problem-solving algorithm (TSP). (**J**) Model smoothed using spline interpolation. (**K**) Distance heatmap from TSP model showing physical distances (in nm) between pairs of beads in the model. (**L**) Distance heatmap from interpolated TSP model showing physical distances (in nm) between pairs of beads in the model. Dashed lines represent loop anchors, (also shown in Fig. 2 M as yellow and teal spheres). (**M**) 3D model of chromatin loop captured in image #17. Beads 1000 (cyan) and bead 2331 (yellow) mark loop anchors. Distance (44.97 nm) between anchors is also shown.

We applied iPALM^60^ to image samples stained with Atto647N tagged oligos, achieving super-resolution imaging (Fig. 1D). In total, we ran ten imaging experiments from which we managed to acquire 25 images for the target chromatin region (Figure S3) which were manually localized and extracted (Figure S4) from multiple nucleus images with various image qualities. Next, we used image information such as XYZ position, uncertainty, and number of photons for voxels containing non-zero iPALM signal (referred to as a peak) in each. For visualization purposes, images were also rendered in 3D. From rendered 3D visualizations (Figs. 1D, 2A and 2B), we observed orderly arranged dots, reflecting the target chromatin conformation.

### iPALM image characterization

To extract loop conformations, we further processed the image data utilizing information exported from raw images. We calculated the number of peaks in each image (Table S3), the average number of photons in each peak (Table S4), the volume occupied by peaks (Table S5, Figure S5), and the distribution of sigma value indicating the uncertainty of voxel position in 3D space (Fig. 2E). The average sigma value allowed us to specify the localization precision of the signal in the image. The best localization precision we managed to achieve in XYZ dimensions was 3 nm × 3 nm × 2 nm respectively (Figs. S6–S8). We also created the 3D visualizations of peaks to observe the distribution of peaks in 3D space (Figs. 2C,F and S9, S10). As we noticed that some peaks show high uncertainty of the position (high sigma value), we filtered out the peaks with high uncertainty value (sigma value higher than 15 nm in all XYZ dimensions). Next, we characterized the number of peaks and their distribution in 3D space (Table S3, Fig. S11 and S12) and the volume by the convex hull (Fig. S13, Table S5) in each image. During iPALM imaging, one oligo produces a signal to generate a cluster of peaks, visualized as a dot in the image. To restore the arrangement of the oligos along the chromatin loop, we then used PeakSelector to generate group peaks from filtered peaks. Grouped peaks represent single oligos of the FISH probe. The distribution of the quantity of the filtered peaks in each group peak is shown in Fig. S14. The average sigma value of X, Y, and Z was also examined to assess the quality of each group peak (Table S6, Fig. S15). The number of group peaks in each image is within the range of the number of oligos in the FISH probe (Table S3), achieving genomic resolution up to 120 bp. For group peaks, the volume was again calculated (Table S5) by convex hull for each image (Figs. 2G and S16). Ultimately, each image (Figure S17, Figure S18) shows one copy of the 33 kb chromatin loop region.

### Reconstruction of the target chromatin loop by iPALM image modeling

Visual verification of the images showed that the images with lower staining efficiency seem to have a more inconsistent distribution of probes, or there seemed to be a part of the target region that was not captured during the imaging (for example images 16, 22, and 24 in the Fig. S18). We assumed that a chromatin region covered in FISH probes entirely can be reconstructed using the shortest path algorithm. Probes located near each other on the fiber connected by the TSP solver algorithm should give us a reliable chromatin loop reconstruction. This assumption was validated by the simulated iPALM analysis (Methods: Simulated iPALM image analysis). By simulated iPALM analysis and visual verification (Fig. S19, S20), we decided to exclude from the further analysis the images presenting the signal from less than 30% of 336 oligos constituting Oligopaint probe, which were visualized as group peaks (Fig. S21). As a result, we obtained 13 good-quality images to proceed with the modeling pipeline (Fig. S22). We further validated our modeling method by verifying how the abscence of a relatively continuous DNA region would influence the reliability of reconstructed structures using random walk polymer simulations and the Modified Jaccard Index^61^. The average IMJ between the reconstructed and the original structure depending on the gap size is presented in Fig. S23. Our observations showed that even when around 70% of input points are missing, the TSP reconstructed models have a higher IMJ score when compared to the original model than the score for random conformations (≈30% as described by Kos et al.). Importantly, our analysis showed that IMJ median values are from around 40% to over 90% (Fig. S23) which is comparable to the values reported by Kos et. al (40–60% IMJ between experimental data and reconstructed models). Therefore we concluded that our modeling method gives reliable results with an even up to 70% of the target region missing from the image. We used the designed image-driven polymer modeling method to simulate the chromatin conformation. In each image, group peaks (Figs. 2C,H and S18) were connected by a traveling salesman problem solver (TSP) (Figs. 2I and S24). The obtained structures were smoothed using cubic spline interpolation to evenly distribute beads along the polymer fiber (Figs. 2D,J and S25). For each model, we assigned the polymer with 3331 beads (10 bp per bead). Our data processing pipeline is shown in Fig. S31. By aligning the genomic locations to each bead in the polymer, we were able to locate the beads that correspond to the loop anchors. Marking the loop anchors in our models allowed us to visualize the loop conformation directly with the ultra-high spatial genomic resolution of 120–300 bp (Figs. 2M and S26). We generated distance maps from each model to describe the contacts within the target chromatin region before (Figs. 2K and S27) and after interpolation (Figs. 2L and S28). We then compared visually the distance maps from interpolated models maps between different images (Fig. S28) and observed significant heterogeneity regarding both loop shape and size in the single cells. We confirmed our observation by calculating the IMJ score^62^ between distance maps from all reconstructed loop conformations. The average similarity was 35% which is close to 30% reported as cell-to-cell variability^62^ (Fig. S29). A similar observation was reported at the scale of TADs by Bintu et al^45^. To validate the use of our Travelling Salesman Problem solver algorithm (TSP), we use the data from multiplexed FISH imaging for chromatin tracing^45^. This method enables the determination of both the genomic coordinates and their 3D structure by imaging multiple 1.2–2.5 Mb regions of human chromosome 21. We took the coordinates of consecutive 30-kb segments in the 2-Mb genomic region (hg38, Chr21:28 Mb-30 Mb) from the cell line of IMR90 lung fibroblasts. Having the coordinates of all the 30-kb segments we then tried to infer the original chromatin structure using our TSP approach. We then compared the inferred order of the chromatin segments with the models from Bintu et al. using Modified Jaccard Index (IMJ)^62^ of the distances between the corresponding segments (Fig. S30). Although the TSP structures did not match the original structures perfectly, they still seem to be similar and the IMJ analysis yielded much better results compared to the random point order. To prove this point we calculated the Spearman's rank correlation coefficient between the correct and inferred orders for around 4,000 structures. The tests were performed with a two-sided alternative hypothesis and statistical significance of 0.05. The correlation was significant in around 82% of the structures and most of them showed extremely low p-value (less than 10^-5). Some of the results from this analysis are presented in Fig. S32.

### High concordance between iPALM imaging and Hi-C or ChIA-PET data

**Figure 3:**
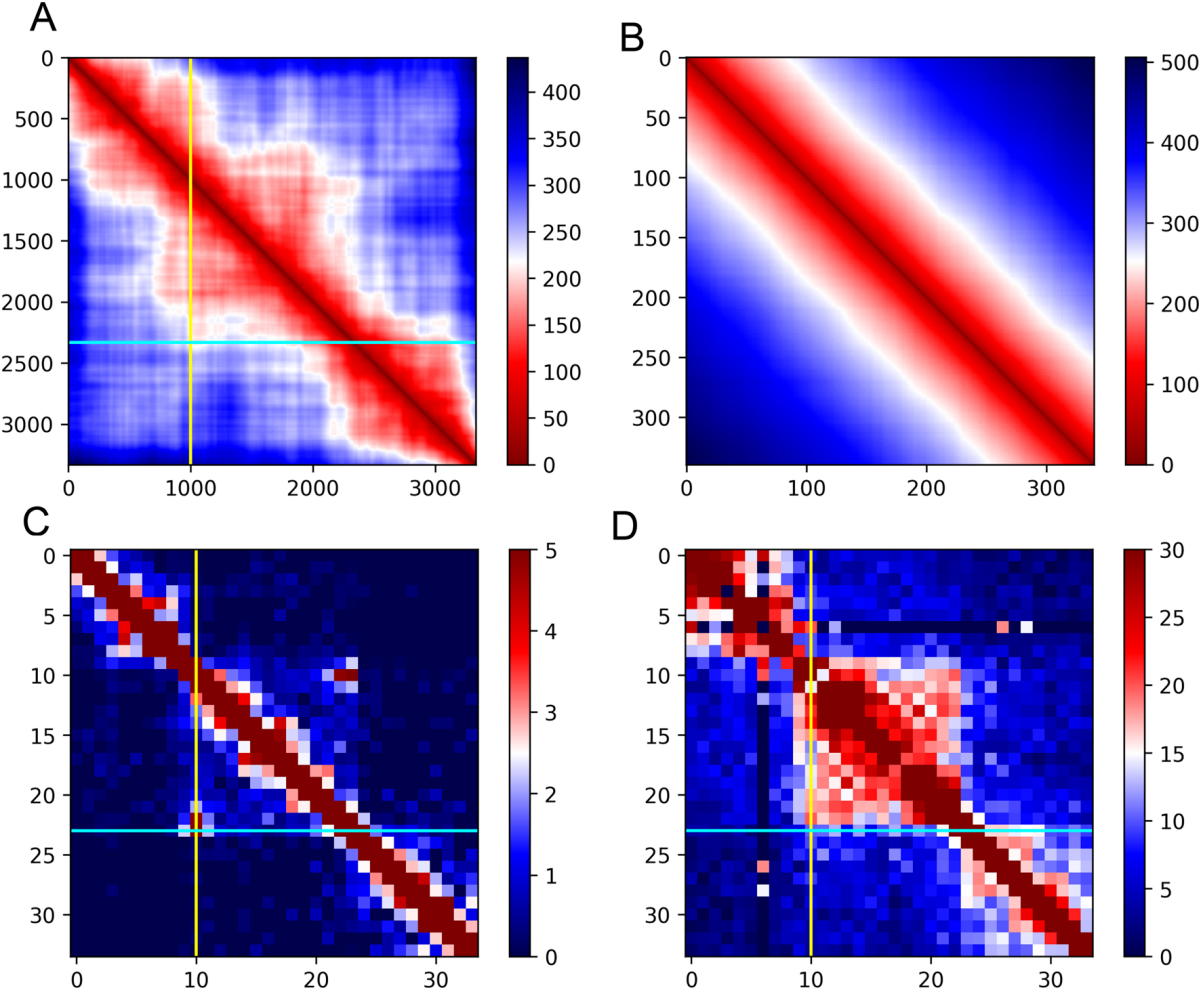
Comparison of average distance heatmaps and genomic interaction heatmaps. Each map shows target chromatin region (chr14:23,016,000–23,049,000). (**A**) Average distance [nm] heatmap calculated from all the 3D models with marked loop anchor positions (yellow line for bead 1000: chr14:23,026,030, cyan line for bead 2331: chr14:23,039,340). (**B**) Average distance [nm] map from simulated control experiment: self-avoiding random walk polymer models. Scale bar in (**A**) and (**B**) represents the linear distance between model beads with red representing the shortest distance and blue the longest. (**C**) ChIA-PET interaction heatmap, 1 kb resolution. (**D**) In situ Hi-C interaction heatmap, 1 kb resolution. Scale bar in (**C**) and (**D**) represents the interaction frequency from ChIA-PET and Hi-C data respectively with red representing the highest frequency and blue the lowest.

**Figure 4:**
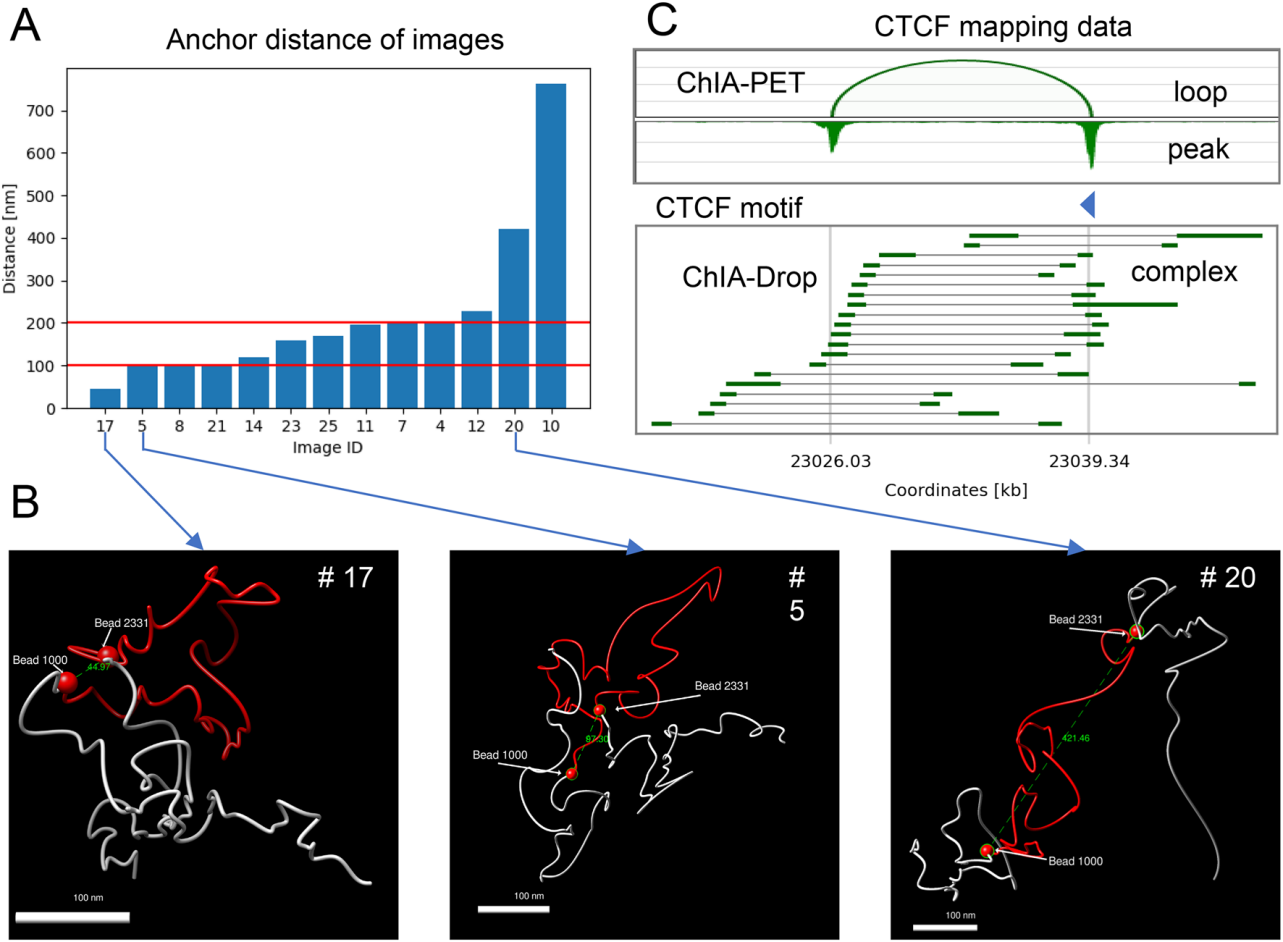
Distribution of loop anchor distances from imaging data-driven polymer model and genomic mapping data. (**A**) Distances between loop anchors from imaging models (**B**) Examples of image models. The loop region is colored in red (chr14:23,026,000–23,039,000). The flanking regions are in grey (chr14:23,016,000–23,026,000, chr14:23,039,000–23,049,000). In each image, the anchors (chr14:23,026,030 and chr14:23,039,340) were marked by red spheres and the distances (in nanometers) are shown in green. (**C**) CTCF ChIA-PET^18^ and ChIA-Drop data (https://data.4dnucleome.org/experiment-set-replicates/4DNESX18HS2V/) at the target loop region. 20 CTCF ChIA-Drop complexes represent the heterogeneity of loop structure in single cells.

We then constructed the average distance map from the 13 interpolated 3D models and compared it to Hi-C and ChIA-PET genomic data on the same genomic region (chr14: 23,016,000–23,049,000). Figure 3A shows the average distance map of our image models. Although not fully restoring ChIA-PET or Hi-C interaction map (Fig. 3C, D), the distance map shows interactions within the loop region as represented in ChIA-PET and Hi-C heat maps. To simulate negative control (the chromatin region without any specific DNA loop) for our experiment we constructed a set of 1000 self-avoiding random walk polymer models. Then we calculated the average distance map of these models the same way we did for image-driven modeling (Fig. 3B). As expected, we observed a high signal between model beads around the diagonal region of the matrix (representing a high frequency of short contact distances) but a low signal in the rest of the matrix (representing long-distance contacts), demonstrating that the genomic features we discovered from image-driven models were not observed in random polymers. Furthermore, we analyzed the loop anchors (beginning and end regions of a chromatin loop overlapping with CTCF convergent motifs and CTCF ChIP-seq peaks locations) positions on 3D target loop models. The exact position of loop anchors was determined by genomic data and then the anchors were imposed on our resulting models. By measuring the physical distance between two loop anchors in the models, we observed that the target loop varies in the anchor distance ranging from 44.97 nm to 761 nm (Figs. 4A,B and S26), suggesting the heterogeneity of target region formation among individual cells. However, the anchor distance is around 100 nm to 200 nm in 10 out of 13 models, while in Image 17 the distance is below 100 nm and in Image 20 and Image 10 it is above 200 nm (Fig. 4A), implying that the probability of the contact between anchors is very high (observed in most cells) although the shape of chromatin loop is heterogeneous (Fig. 4B). Interestingly, from publicly available CTCF ChIA-Drop data (https://data.4dnucleome.org/experiment-set-replicates/4DNESX18HS2V/), we also observed the contact variation at this target region (Fig. 4C). ChIA-Drop is a single molecule mapping technique that can uncover multiplex chromatin interaction using droplet-based and barcode-linked sequencing method^63^.

To compare our results with higher resolution genomic data we processed publicly available GM12878 Micro-C data from Dovetail Genomics. We extracted the interactions from our region of interest and visualized the interaction map in three different resolutions: 150 bp (the highest resolution that can be achieved using Micro-C^64^, Fig. S32A), 500 bp (Fig. S32B), and 1000 bp (Fig. S32C). We then compared the interaction maps with Hi-C data obtained for the same cell line by Rao 2014^54^ (Figs. S32D and 3D). Visual inspection indicates that the general structure of the region is similar in Micro-C and Hi-C maps, although in case of the Micro-C maps the signal density is much lower, suggesting that 800 million of reads does not provide sufficient sequencing depth to obtain ultrastructural details of our region of interest (Fig. S32).

## Discussion

This study focused on the use of iPALM microscopy to visualize a 13 kb chromatin loop (and 10 kb flanking regions on each side) detected previously by ChIA-PET and Hi-C. The novel approach consists of the combined use of FISH staining of a very short (33 kb) chromatin fragment, iPALM microscopy, and TSP-based loop reconstruction. According to our knowledge, this allowed us to obtain higher three-dimensional resolution than models generated using: (1) widely available population genomic-based data-driven modeling such as Hi-C, or ChIA-PET and (2) available imaging-based chromatin structure reconstruction models. We believe that by combining iPALM microscopy and Travelling Salesman Solver we generated models of the single chromatin loop with unprecedented resolution. Obtaining both high microscopic localization precision (between 4 and 22 nm) and genomic resolution (120 bp to 300 bp), we achieved single-molecule tracking, allowing for modeling the folding of 10 nm nucleosome fiber near the TCRA locus in human chromosome 14.

Recent studies in targeted chromatin imaging have made significant progress in higher-order chromatin structures identification by applying Oligopaints-based DNA staining and super-resolution microscopy. Up to now most of them focused on large-scale regions such as megabase-size TAD domains, or hundreds of kilobase sub-TAD domains^43–45,47,53^. Barton et al.(2018) presented an conceptually similar to iPALM approach called ChromoTrace^65^. The main difference between them is the use of different imaging techniques. In our case, we use one color staining. ChromoTrace aims to reconstruct the structure of whole chromosomes using the suffix trees approach based on at least 4 color chromatin staining. It is purely theoretical and based solely on simulated datasets method. The resolution of both approaches is different as well. We try to identify every single probe attached to the DNA fiber which makes our ideal resolution around 100 bp. ChromoTrace aims at 10 kb resolution. This makes the resolution of our approach much higher, but also more sensitive to staining efficiency.

Notably, Mateo et al. has visualized the regulatory chromatin folding at the Drosophila BX-C region by a novel method of the optical reconstruction of chromatin architecture (ORCA)^53^, getting genomic resolution up to 2 kb. In our study, we resolved a detailed structure of a single chromatin loop with a higher genomic resolution of 120–300 bp by modeling the 10 nm fiber folding. We processed the images to precisely estimate the positions of oligoprobes attached to chromatin fiber. We also verified our approach by applying the modeling pipeline to simulated structures and proved that we can reconstruct the original shape of the loop structure if the coverage of chromatin with oligo probes is high enough.

Initially we expected to see a single looping structure in the target chromatin region as inferred by population-averaged ChIA-PET and Hi-C genomic data, however, in super-resolution imaging we observed more heterogeneous folding conformation in each image, implying that the chromatin looping is affected by other factors (like nucleosome stacking and cohesin-mediated extrusion) besides the architectural CTCF proteins bound on both anchors. LE model provides one of the mechanisms that could account for the variety of loop forms that we observe using the iPALM method. We hypothesize that different loop structures that were captured microscopically in our study might be a representation of different stages of the LE process. For example in one of the loop models (model #20 Fig. S25, S26) we can see a very small loop that could correspond to the beginning of the LE process. We speculate that having a larger number of good quality loop images would allow us to reconstruct the course of LE in the cells. Additionally, studied loop is localized within open, accessible chromatin region within A compartment (subcompartment A1), which suggest low DNA density and looser stabilization of chromatin structure that could also contribute to the heterogeneity of loop structure observed in our study.

Importantly, regardless of the differences between methods in characterizing single cells and population cells, we observed the concordance between microscopic visualization and genomic characterization presented by the average distance map and interaction maps from ChIA-PET and Hi-C, respectively, suggesting the validity of our imaging method and consistency of obtained models and genomic data for this chromatin region. Comparison of our data with ultra-high-resolution 3D genome architecture maps obtained using Micro-C approach also showed a visual similarity between both Hi-C interaction map and distance map from our models However relatively low sequencing depth of this experiment made it impossible to effectively compare high-resolution image-driven models. We believe that in order to reliably compare our distance maps obtained from 3D models at around 150 bp resolution with Micro-C matrices, the sequencing depth of the Micro-C dataset would have to be much higher to reflect the fine structure of the region. We see Micro-C as an interesting and promising approach for studying the detailed structure of chromatin fiber. Nevertheless, an important drawback of this method is that it requires extremely high sequencing depth, which is associated with very high costs of experiments.

We also calculated a set of self-avoiding random walk models and compared the average distance map with our results. As expected, the structures derived from image-driven models of the target chromatin region are not present in control chromatin structures generated using a random walk model. Comparably, the heterogeneity of chromatin interaction within the same region was also observed by ChIA-Drop which captures chromatin contact complexes with single-molecule precision. We observed high structural heterogeneity. We expected to clearly see chromatin looping (Fig. 1D and 4B) in the middle of the studied region in most of the images. However, we learned that single models don’t always reflect the structure suggested by population genomic data (Fig. 1A and 3C,D). Distance maps from single models do not look like Hi-C or ChIA-PET interaction maps, but strikingly it is enough to average the distances from thirteen different image-driven models to observe the similarity to population genomic-driven data (Fig. 4A). To verify whether our modeling method can be used in other datasets we tested it on the cryosection-based method (3D EMISH^47^). Restoring chromatin shape from density-based images requires a modification of our modelling pipeline to include the information about the density field into the method, and to avoid creating edges that extend beyond the image area. We are currently working on the optimization of this method. We also tested our approach on the Bintu et al. data set^45^ and proved that we can reconstruct the chromatin shape from different microscopic approaches and on different scales.

Collectively, our study provided novel insights into the nature of chromatin folding in vivo, despite the limitations of the experiment. Combining multi-color sequential staining^43,45,53^ with iPALM imaging, would be a promising approach to recovering single loop structure without the use of statistical modeling. Interestingly, recently new imaging technologies such as Minflux^66^, SIMflux^67^, or 4pi-SMLM^68^ were reported. They aim at improving the localization precision of the probes and the resolution of the images while limiting the effects of fixation on the biological samples. While those methods still have limitations, we recognize them as promising techniques for studying the loop structure in 3D intact nuclei followed by image-driven DNA structure modeling in the future.

Recently the effective staining of the chromatin poses even bigger challenges than increasing the resolution of microscopy technologies^38^. The exact effect of the fixation of the DNA and staining of the fiber with oligoprobes is still not well understood. In this regard Hobro et al. studied the effects of different fixation methods and compared the images to Raman spectroscopic imaging of live cells to assess the impact of fixation on the quality of images^69^. Aldehyde fixation methods performed better and cause less severe loss of biochemical information than organic solvents. Of note, to better understand the effects of staining on chromatin structure CRISPS-Cas9-mediated in situ labeling of genomic loci could be used^70^. In contrast to classical FISH, this method does not require DNA denaturation thus permitting better structural preservation of the sample. This would allow observing the structural differences between chromatin samples stained with classical FISH staining and with CRISPS-Cas9-mediated in situ labeling and assessing the effect of hybridization of chromatin 3D structure.

The key development in fluorescent microscopy would be to minimize the potential changes in DNA shape during experiments. One potential solution would be to perform the DNA denaturation in situ for example by chemically freezing the chromatin, which decreases its flexibility. Such a procedure however would require careful optimization. The main challenge lies in the fact that the attachment of oligoprobes to the DNA requires a relatively flexible DNA double helix therefore the chemical freezing of chromatin cannot be too strong. Additionally, decreasing the time of the sample preparation would also be beneficial for preserving the DNA structure by avoiding DNA denaturation. Progress in chromatin staining in live imaging is another approach to minimize the interference in chromatin structure during the experiment^71,72^. For example, Gu and colleagues managed to measure the movement of enhancers and promoters in live embryonic stem cells by combining a molecular assembly strategy termed chimeric array of gRNA oligonucleotides (CARGO) with dCas9 (catalytically dead Cas9) imaging^73^.

Although we have implemented negative controls by image computational modeling, this could also be done experimentally by microscopic observation of the non-looping region to explicitly address the differences between looping and non-looping conformations. In addition, imaging various scales of single chromatin loops is another topic for us in the near future. Besides small-scale chromatin loops, hundreds of kilobase sub-TAD and megabase TAD could also potentially be imaged and modeled for their detailed structures.

Another significant development would be to directly observe the positioning and organization of the nucleosomes as Ohno et al. did using population genomic methods^74^. This would require further improvement of microscopic technologies and would allow us to build very detailed models and include the information about the thickness and packing of chromatin and flexibility of the fiber in our single-cell models.

It was proposed that chromatin flexibility could be an important factor in chromatin structure regulation^75^. Using simulations of nucleosomes connected by flexible DNA segments it was shown that small local changes in histone H1 positioning can affect chromatin flexibility and that it might influence the establishment of long-range chromatin interactions^76^. Generally, the lower the local nucleosome concentration, the higher the chromatin flexibility. Importantly it is known that protein flexibility facilitates protein-DNA interactions^77^. Similarly, we hypothesize that increased DNA flexibility could also help in its binding with proteins. With the improvement of staining and microscopy methods our approach could be applied and further developed to discover novel physical properties of chromatin structure in single cells.

## Methods

### Cell culture

Human GM12878 cells were cultured in RPMI 1640 with 15% fetal bovine serum at 37 °C and ambient 5% CO_2_ as described by the Coriell Institute of Medical Research. Cells at the exponential growth phase were harvested for nuclei isolation. All the cells used in this study were negative for mycoplasma.

### Nuclei and coverslip preparation

Considering the limited penetration depth (500–700 nm) of interferometric photoactivated localization microscopy (iPALM) compared to micrometer cells, we isolated the nuclei to avoid imaging the target region through the cytoplasm. Nuclei were isolated from GM12878 cells by using Nuclei EZ Prep Nuclei Isolation Kit (Sigma) which was previously applied for visualization of chromatin in isolated nuclei. This kit was developed to study chromatin, histones, and nuclear RNA/RNP and has been widely applied including in chromatin imaging^78^. The whole procedure was done at 4 °C within an hour. The integrity of isolated nuclei was confirmed by inspection under a conventional microscope. Coverslips with gold nanorods (see in “Materials Methods” of “iPALM imaging”) were incubated in 1 M KOH for 20 min, washed with water, coated with 0.01% poly-L-lysine (Sigma) for 20 min, rinsed with water, dried for 30 min for later use.

### DNA FISH

Isolated nuclei were added to attach to poly-L-lysine coated coverslips, fixed with 4% paraformaldehyde for 10 min at room temperature, rinsed with 1 × PBS, permeabilized with ice-cold methanol for 10 min, rinsed with 1 × PBS, dehydrated with 75%, 85%, and 100% ethanol series for 2 min each, dried at 60 °C for 1 h. Oligopaint FISH probe (Arbor Biosciences) was mixed with DNA hybridization buffer and added to prepared nuclei, denatured at 80 °C for 5 min, incubated in a humid chamber at 37 °C overnight. The next day, the nuclei were washed with 50% formamide in 2 × SSC at room temperature for 10 min, followed by a further wash with 2 × SSC at room temperature for 10 min, 0.2 × SSC at 55 °C for 10 min, and then a large volume of 2 × SSC at room temperature till imaging.

### iPALM imaging

Samples were imaged in standard stochastic optical reconstruction microscopy (STORM) buffer by iPALM^60^. Isolated nuclei were adhered to 25 mm round coverslips containing gold nanorod particles that act as calibration standards and alignment/drift fiducial markers. These were prepared as described in^79^. Briefly, coverslips were washed for 3 h at 80 °C in a 5:1:1 solution of H_2_O:H_2_O_2_:NH_3_OH, rinsed copiously and coated with poly-L-lysine. After further washing, gold nanorods (Nanopartz, Inc) were adhered to poly-L-lysine coated coverslips, washed again, and coated with ca. 50 nm SiO_2_ using a Denton vacuum evaporator. Samples were mounted in dSTORM buffer^80^, containing tris buffered saline, pH 8, 100 mM mercapto ethanolamine, 0.5 mg/mL glucose oxidase, 40 ug/mL catalase, and 10% (w/v) glucose (all from Sigma). An 18 mm coverslip was adhered atop the bottom coverslip, sealed, mounted in the iPALM, and imaged as described above.

Briefly, samples were imaged at 30–50 ms exposure, under 3 kW/cm^2^ of 647 nm laser excitation and 100 W/cm^2^ 405 nm laser activation for 25,000 frames to capture blinking Atto647N molecules. Data were imported into the PeakSelector software package (Janelia Research Campus), which registers three camera images with respect to each other, calibrates the intensity across each camera as a function of z-position, and localizes each blinking molecule in 3D. Further, fiducial nanoparticles embedded in the coverslip allow for drift correction after acquisition and localization to maximize image resolution. After the processing and drift correction, spatial positions were filtered based on localization uncertainty in all three dimensions (XYZ).

### Image pre-processing

iPALM images were reconstructed via localization of blinking fluorophores over 25,000 frames across each of three EMCCD cameras. Gold nanoparticles act as fiducial markers that allow for (1) spatial registration of the three cameras using a full affine transformation and (2) calibration of the z-position response of the system. After localization, images were filtered to only include localizations with < 30 nm uncertainty in all three dimensions. The gold fiducial particles also allow for drift correction in all three dimensions, and for correcting any sample tilt to within 30 nm error. iPALM data were rendered in 3D using PeakSelector (Gleb Shtengel and Harald Hess, Howard Hughes Medical Institute, https://github.com/gleb-shtengel/PeakSelector) as described in (Shtengel et al. 2009^60^ and Sage et al. 2015^81^).

### Multidimensional scaling

Multidimensional scaling (MDS) algorithm is a statistical method which takes a matrix of similarities or distances between objects and put those objects in N-dimensional space possibly close to given distances^82^. We can use Hi-C/ChIA-PET relative frequency contact matrix into physical distances and seek its representation in 3D space^83^. This matrix is interpreted as a graph neighborhood matrix. Using this graph, we calculated graph distance. The result was an input to the MDS algorithm (Scikit implementation^84^). In this case, every bin in the interaction matrix will represent a single bead in a model in 3D. We used a Hi-C contact matrix for the studied region to obtain a set of 300 3D chromatin polymer models.

### Travelling Salesman algorithm

The traveling salesman problem is an NP-hard problem that, given a set of points in space, finds the shortest path that visits all of them. We can assume that in the case of iPALM images the probes attaching to the chromatin fiber should form such a path and reconstructing it would give a possible shape of the DNA loop. It represents the lowest complexity computational model, given the set of points. We used the implementation of the greedy algorithm to find one of the best solutions for this problem (https://github.com/dmishin/tsp-solver). We treat our set of points as graph nodes and distances between them as vertices. At the beginning, each vertex is a separate path of length 1. In each step, we are finding two closest disconnected paths, and we connect them into one. This step is repeated until there is just one path left. Greedy TSP solver gives highly non-optimal results therefore after connecting all paths into one we run an optimization algorithm. Optimization tries to rearrange dots in the path to improve the solution. After finding the shortest path we are simply deleting the longest connection between two dots. This way we get the shortest path between two dots that are the furthest from each other. The algorithm was used to model all 13 good quality target loop images.

### Spline Interpolation

We used cubic spline interpolation to smooth structures modeled from iPALM images. Our implementation also allowed us to distribute model beads evenly along the polymer fiber, which guaranteed that each bead of the model represents an equal fragment of chromatin fiber. We interpolated each model to 3331 beads. As the target region was 33 312 base pairs it gave us the simulated genomic resolution of 10 bp per bead of the computational model, which allowed for the exact localization of loop anchors on the polymer model.

### Simulated iPALM image analysis

We simulated a set of 300 Hi-C data-driven models from the studied region using a Multidimensional scaling approach^82^. Simulated models were rescaled to fit the spatial size of images obtained from the iPALM experiment. Each model was interpolated to 340 points (beads of the model). This way each bead roughly corresponds to the region that would be covered by a single FISH probe (~ 100 bp). From each simulated model we randomly chose subsets of original beads. Each subset corresponds to the different staining efficiency (5% probes attached, 10% probes attached, … , 95% probes attached). Each set of beads was then reconstructed by our modeling procedure (TSP solver) and interpolated to 340 beads. This way for each simulated structure we got a set of reconstructed models that could be compared to the original. We aligned those structures (examples are shown in Figs. S19 and S20). Based on this analysis we concluded that TSP indeed seems to reconstruct the original polymer structure, but we need to see at least 30% of the original probes in the iPALM image for our procedure to maximize the liability of the results.

### Gap size influence on reconstruction accuracy

We studied the effect of a lack of staining for a relatively continuous DNA region by performing a simulation of a set of 20 self-avoiding random walk structures via structure Generator Python software (https://bitbucket.org/4dnucleome/structuregenerator/). Each structure consists of 340 beads. From each simulated structure, we randomly chose a gap of consecutive beads of a given size. We were not able to assess the size of gaps in iPALM experimental data as we did not have the information on which probes failed to attach to the target region. The images that we accepted for loop reconstruction had a minimal number of input points equal to ~ 33% of introduced oligoprobes. This means that the maximal possible (but not likely as the rest of the region would have to be perfectly covered by oligoprobes) size of the gap is equal to 230 points. To overcome the fact that we are not able to precisely assess the size of missing points within a region in our images we extended the range of the gap size from 1–100 points to 1–230 points (out of 340 original data points in the input random walk model), which allowed us to study the influence of all possible gap sizes on structure reconstruction precision. For each gap size and each original random walk structure, we calculated 10 “leaky” structures with a single random gap of a given size. Each of the original sets of beads was then reconstructed from each of our “leaky” structures by our modeling procedure (TSP solver) and interpolated to 340 beads. Then we used the Modified Jaccard Index (IMJ)^61^ to compare the distance maps of these structures with the distance map of the original model (Fig. S23).

### Image generation and analysis pipeline

Experimental steps were performed to select the target region based on genomic data, design an Oligopaint probe, perform FISH staining followed by iPALM imaging. Next, we preprocessed the images in PeakSelector software and exported the peak localization information in ASCII format. The preprocessed data were analyzed (calculation of the peak location uncertainty, peak quality, and image volume), filtered, and clustered. After the image quality assessment, the x, y, z positions of clustered group peaks were exported to PDB format and used to reconstruct the target chromatin loop by the TSP algorithm and spline interpolation). The results were visualized in Chimera software^85^. Image model validation and 3D model analysis consisted of calculation of the distance map for each model, localization of loop anchors on 3D models, and comparing the results with genomic data (ChIA-PET and Hi-C). The analysis pipeline is presented in Fig. S31.

### Micro-C analysis

We took advantage of publicly available GM12878 Micro-C data from Dovetail Genomics, a leading commercial provider of 3C technologies including Micro-C, (https://micro-c.readthedocs.io/en/latest/data_sets.html). Dovetail Micro-C Kit uses the Micrococcal nuclease (MNase) enzyme instead of restriction enzymes for chromatin digestion. This results in evenly distributed fragments of 146 bp. We chose the largest dataset available (800 million sequenced reads) to achieve the highest resolution possible. The data were processed using methods recommended by Dovetail (https://micro-c.readthedocs.io/en/latest/before_you_begin.html) and produced a .hic file using Juicer tools^86^ (https://github.com/aidenlab/juicer). We extracted the interactions from our region of interest and visualized the interaction map in three different resolutions.

## Supplementary Information

Supplementary Information 1.

Supplementary Information 2.

Supplementary Information 3.

Supplementary Video 1.

## Data Availability

All 25 images obtained during the iPALM experiment, and all resulting models are publicly available at https://github.com/SFGLab/ChromoLooping/tree/main/data.

## Code availability

Code for data analysis and modeling together with examples of usage is publicly available at https://github.com/SFGLab/ChromoLooping.
