# Publisher Correction: Super‑resolution visualization of chromatin loop folding in human lymphoblastoid cells using interferometric photoactivated localization microscopy

**DOI:** 10.1038/s41598-022-26502-6

**Published:** 2022-12-20

**Authors:** Zofia Parteka-Tojek, Jacqueline Jufen Zhu, Byoungkoo Lee, Karolina Jodkowska, Ping Wang, Jesse Aaron, Teng-Leong Chew, Krzysztof Banecki, Dariusz Plewczynski, Yijun Ruan

**Affiliations:** 1 The Jackson Laboratory for Genomic Medicine, 10 Discovery Drive, Farmington, CT 06030 USA; 2 Centre of New Technologies, University of Warsaw, S. Banacha 2c, 02-097 Warsaw, Poland; 3 Faculty of Mathematics and Information Science, Warsaw University of Technology, Warsaw, Poland; 4 Department of Genetics and Genome Sciences, University of Connecticut Health Center, 400 Farmington Avenue, Farmington, CT 06030 USA; 5 Centre for Advanced Materials and Technologies, Warsaw University of Technology, Poleczki 19, 02-822 Warsaw, Poland; 6 Advanced Imaging Center, Janelia Research Campus, Howard Hughes Medical Institute, 19700 Helix Drive, Ashburn, VA 20147 USA

Correction to: *Scientific Reports* 10.1038/s41598-022-12568-9, published online 20 May 2022

The original version of this Article omitted affiliations for Dariusz Plewczynski. The correct affiliations are listed below.

The Jackson Laboratory for Genomic Medicine, 10 Discovery Drive, Farmington, CT, 06030, USA

Centre of New Technologies, University of Warsaw, S. Banacha 2c, 02-097, Warsaw, Poland

Faculty of Mathematics and Information Science, Warsaw University of Technology, Warsaw, Poland

The original Article has been corrected.

