# Supplementary material for: Super-resolution visualization of chromatin loop folding in human lymphoblastoid cells using interferometric photoactivated localization microscopy
Source: Sci Rep. 2022 May 20;12:8582. doi: 10.1038/s41598-022-12568-9 (PMC9122977; doi:10.1038/s41598-022-12568-9)
Supplement: Supplementary file 1 — Supplementary Information 1. [file 41598_2022_12568_MOESM1_ESM.pdf]

README.md

ChromoLooping

Code and data in this repository comes from publication *Super-resolution visualization of chromatin loop folding in human lymphoblastoid cells using interferometric photoactivated localization microscopy*

You can use UCSF Chimera or any other viewer to visualize models and points in .pdb format.

Installation Guide

Requirements for Python packages can be found in **requirements.txt**

Requirements:

- Python 3.6 or later
- Jupyter Notebook
- UCSF Chimera or other visualization software

The installation on desktop computer shouldn't take longer than 30 minutes.

The easiest way to run the code is to open Jupyter notebooks:

```
$ git clone https://github.com/SFGLab/ChromoLooping.git
$ cd ChromoLooping
$ jupyter notebook
```

...and select one of the two available **.ipnyb** files:

- statistics and image data processing
- modelling pipeline

The existing examples in Jupyter notebooks can also be viewed directly on GitHub.

Jupyter Notebooks

All examples of usage of the full processing pipeline step by step can be found in Jupyter notebooks. In notebook **image_statistics.ipynb** you can find all the operations on ASCII files with image peaks exported from PeakSelector:

- reading and processing iPALM data from ASCII files
- statistics
- plotting histograms
- plotting convex hull

Basic statistics for single iPALM image

In [4]:
```
stats.print_statistics(image=image)
```

```
33292185.477251135
639699.1535913193
Group Count:  115
Peak Count:   1483
Average peaks per group   12.895652173913044
Average sigma X:  9.152259705196006 nm
Average sigma Y:  13.023146327283655 nm
Average sigma Z:  5.397415140616225 nm
Convex hull volume:   33292185.477251135
Convex hull area:    639699.1535913193
Average number of photons per peak:  9534.12137559002
Average number of photons per group peak: 146923.75652173912
Average sigma X per group peaks: 6.232244802608697 nm
Average sigma Y per group peaks: 6.549014719130437 nm
Average sigma Z per group peaks: 3.302540886956522 nm
```

3D plots

3D plots (peaks in 3D space and convex hull)

- all peaks - all blinking voxels recorded by iPALM microscopy
- filtered peaks - blinking voxels filtered by sigma parameter (peak position uncertainty)
- group peaks - detected oligo probes (after peaks clustering and choosing a representat for each cluster)

Convex hull volume is shown in nm^3

In [5]:
```
# all peaks
peaks = PeakPositions(image=image, sigma_threshold=None, minimize=True)
peaks.plot_peak_positions(title="All Image Peaks", outpath=None)
peaks.plot_convex_hull(title="All Peaks Convex Hull", outpath=None)
```

In **image_driven_modeling.ipynb** we put whole modeling procedure:

- exporting image peaks to .pdb format
- Traveling Salesman Problem solver
- Spline interpolation
- Calculating distance maps from 3D models
- Distance maps visualization

Travelling Salesman Problem solving algorithm run on points in .pdb format saved from images files

Saving peaks in .pdb format

In [1]:
```
from peak_stats.reader.peaks import Image
from peak_stats.reader.read_peaks import Reader
from peak_stats.export_pdb.ascii_2_pdb import Points
from peak_stats.statistics.single_img_stats import GroupPeakStats
```

In [2]:
```
ascii_file="data/ascii_files/Run1-647_c123_sum_X5_processed_jsa_IDL_ASCII_image21.txt"
```

In [3]:
```
pdb_file = "data/example_image21/image21_group_peaks.pdb"
```

In [4]:
```
reader = Reader(ascii_file)
image = Image(reader)
img_peaks = Points(image=image)
group_peaks = GroupPeakStats(image=image)
img_peaks.save_pdb(filename=pdb_file, points=group_peaks.positions)
```

Travelling Salesman Problem solver

Run the Greedy TSP Solver on set of points in 3D space to find the shortest path connecting all points.

In [5]:
```
from modeling_scripts.modeling_operations.create_structure import load_points, save_pdb
from modeling_scripts.modeling_operations.create_structure import run_image_tsp
from scipy.spatial.distance import squareform, pdist
```

In [6]:
```
tsp_pdb = pdb_file[:-4] + "_tsp.pdb"
in_points = load_points(infile=pdb_file)
graph = squareform(pdist(in_points))
arranged_points = run_image_tsp(graph=graph, points=in_points, endpoints=None, optimization_steps=10)
save_pdb(points=arranged_points, filename=tsp_pdb, connect=True)
```

```
File data/example_image21/image21_group_peaks_tsp.pdb saved...
```

Out[6]:
```
'data/example_image21/image21_group_peaks_tsp.pdb'
```

This tasks do not require any heavy computations and running the examples in Jupyter notebooks shouldn't exceed 5 minutes on a standard desktop computer.

Data

The results from our work can be fully reproduced using the code from this repository.

All data files used in our publications are located in `data/ascii_files` .

All models obtained from our iPALM imaging are in `data/3d_models` . The models are in .pdb format and can be opened via USCF Chimera software.

example:

```
$ cd data/3d_models
$ chimera image7_smooth3331.pdb
```

The model will look like this:

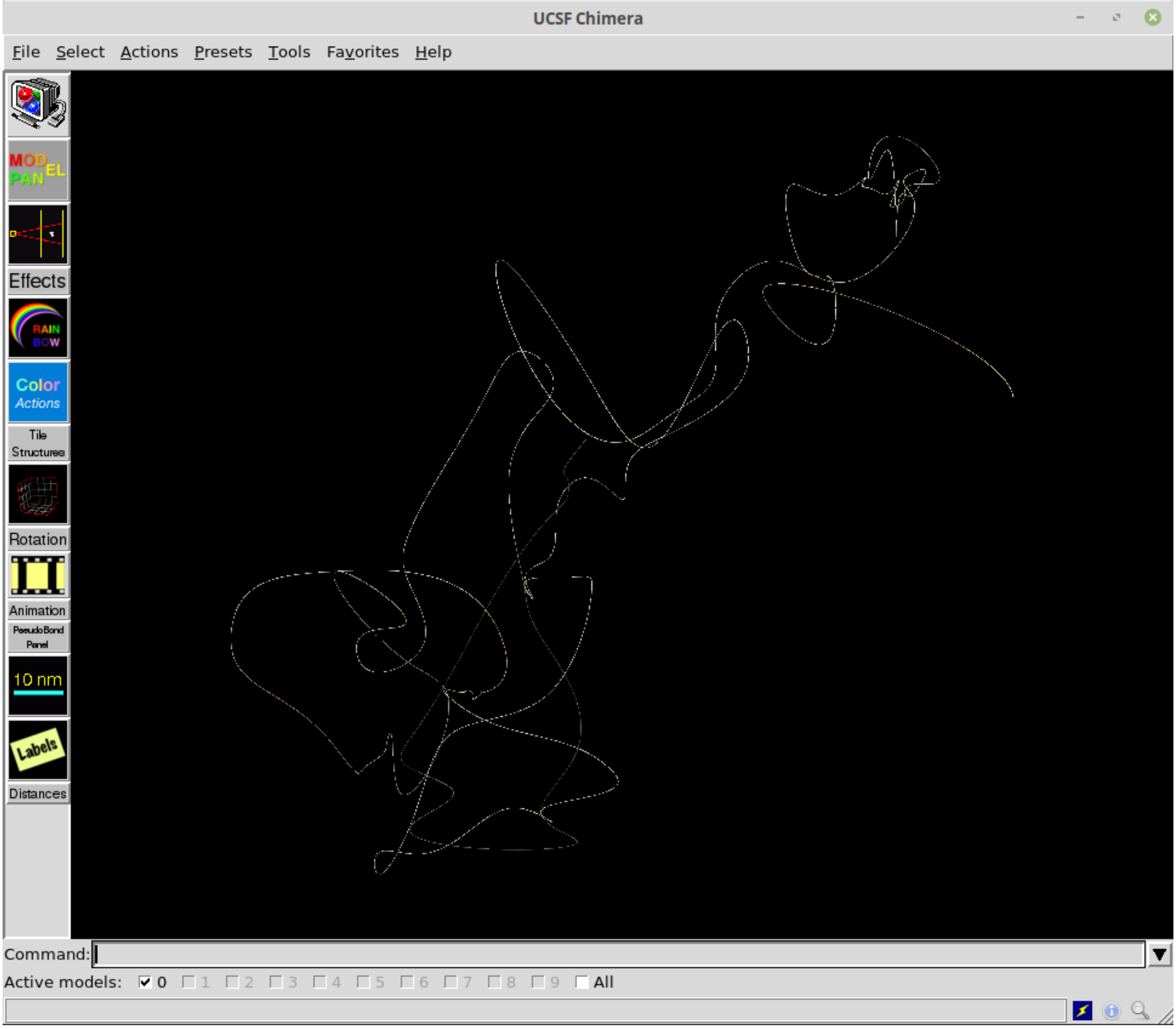

To get the presentation of the model as in our publication open the command line `Favorites > Command Line` and type two commands:

```
setattr m stickScale 8
rainbow
```

Then you can rotate the model to desired orientation. The final result will look like this:

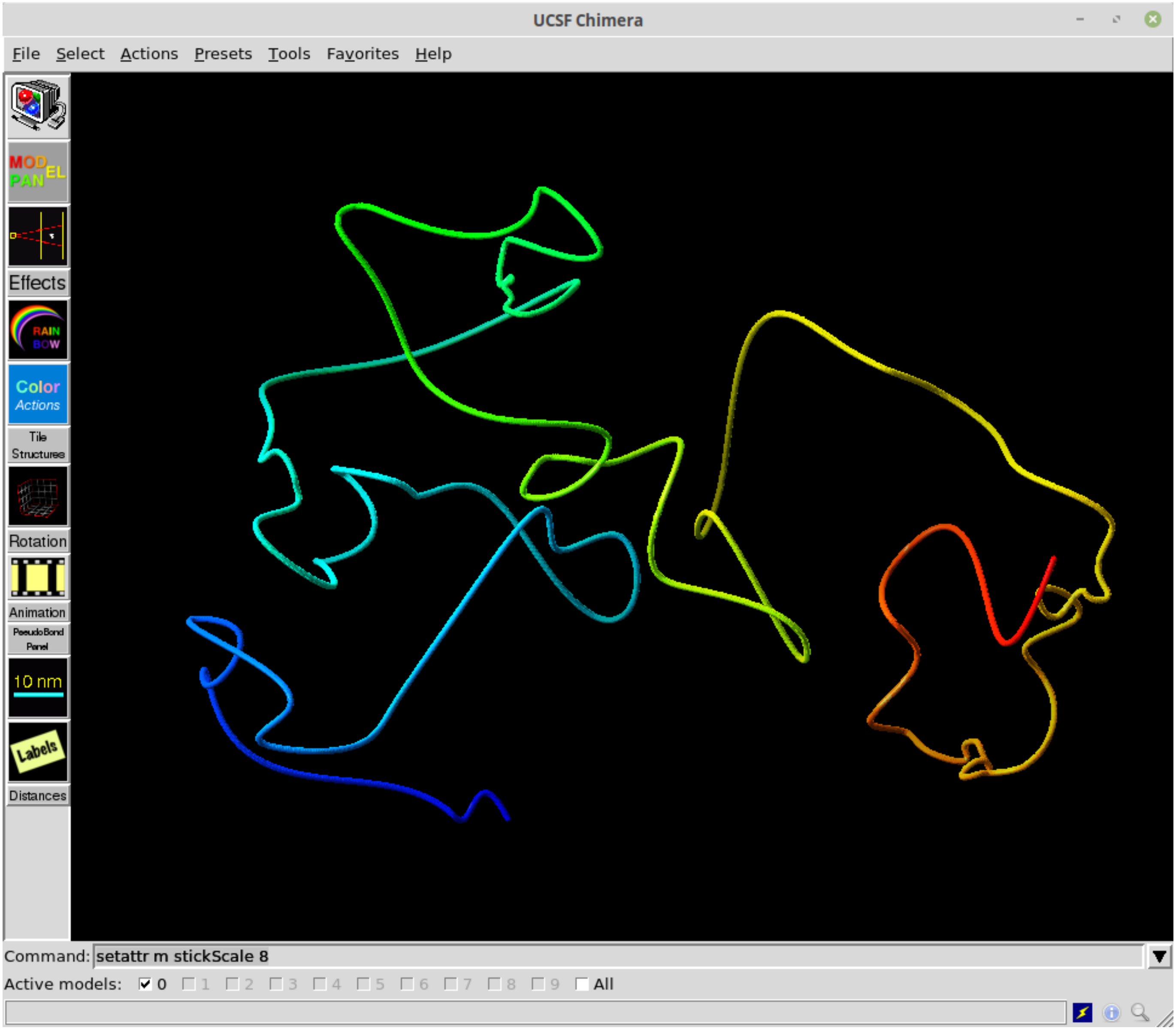

The example output data of full reconstruction of the image processing and modelling pipeline is located in `data/example_image_21` .
