# Supplementary figures and images for: Super-resolution visualization of chromatin loop folding in human lymphoblastoid cells using interferometric photoactivated localization microscopy
Source: Sci Rep. 2022 May 20;12:8582. doi: 10.1038/s41598-022-12568-9 (PMC9122977; doi:10.1038/s41598-022-12568-9)

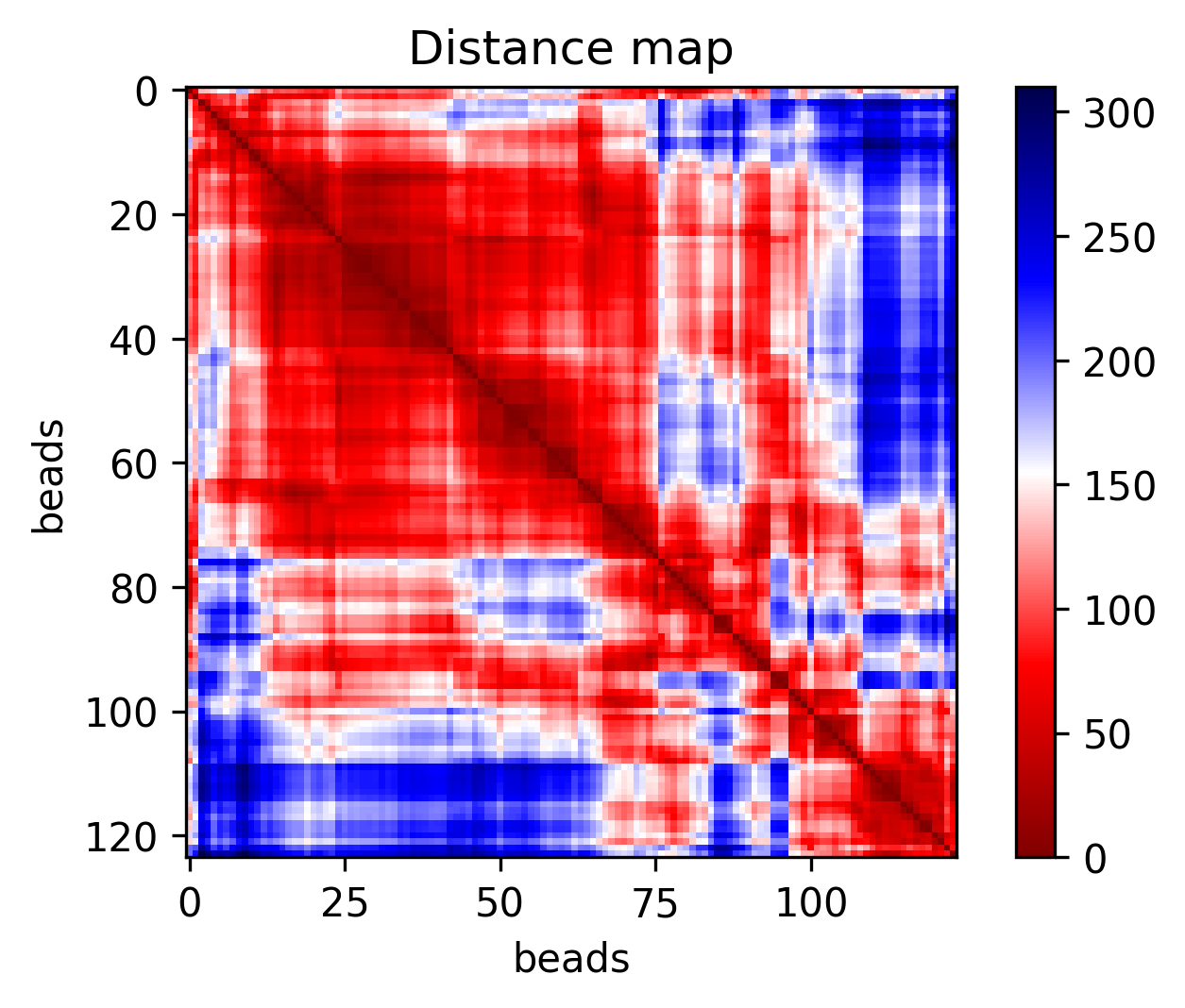

Supplement: Supplementary file 2 — Supplementary Information 2. [file 41598_2022_12568_MOESM2_ESM.zip › ChromoLooping-main/data/example_image21/image21_group_peaks_tsp_seismic_r.png]

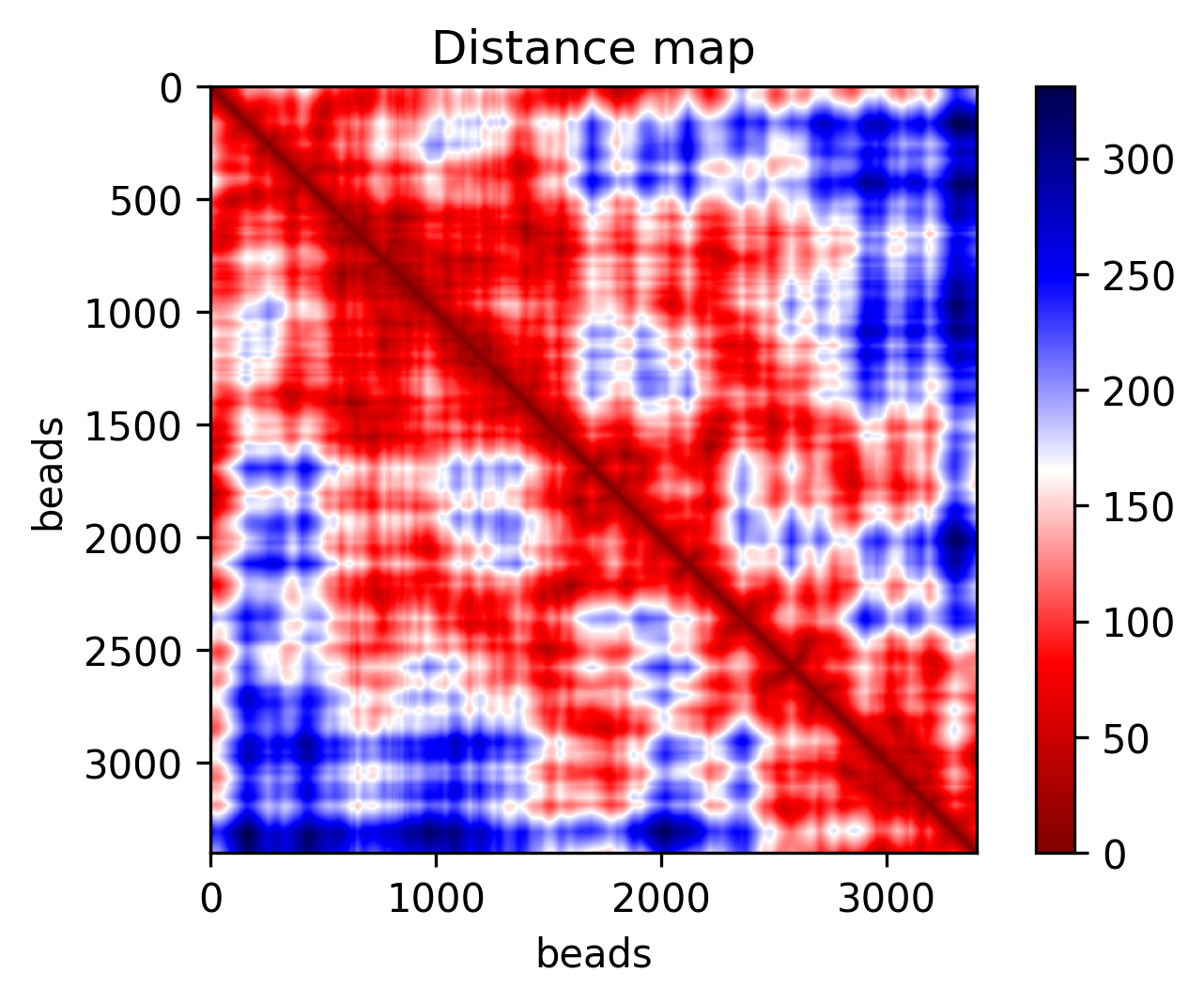

Supplement: Supplementary file 2 — Supplementary Information 2. [file 41598_2022_12568_MOESM2_ESM.zip › ChromoLooping-main/data/example_image21/image21_group_peaks_tsp_smooth3400_seismic_r.png]

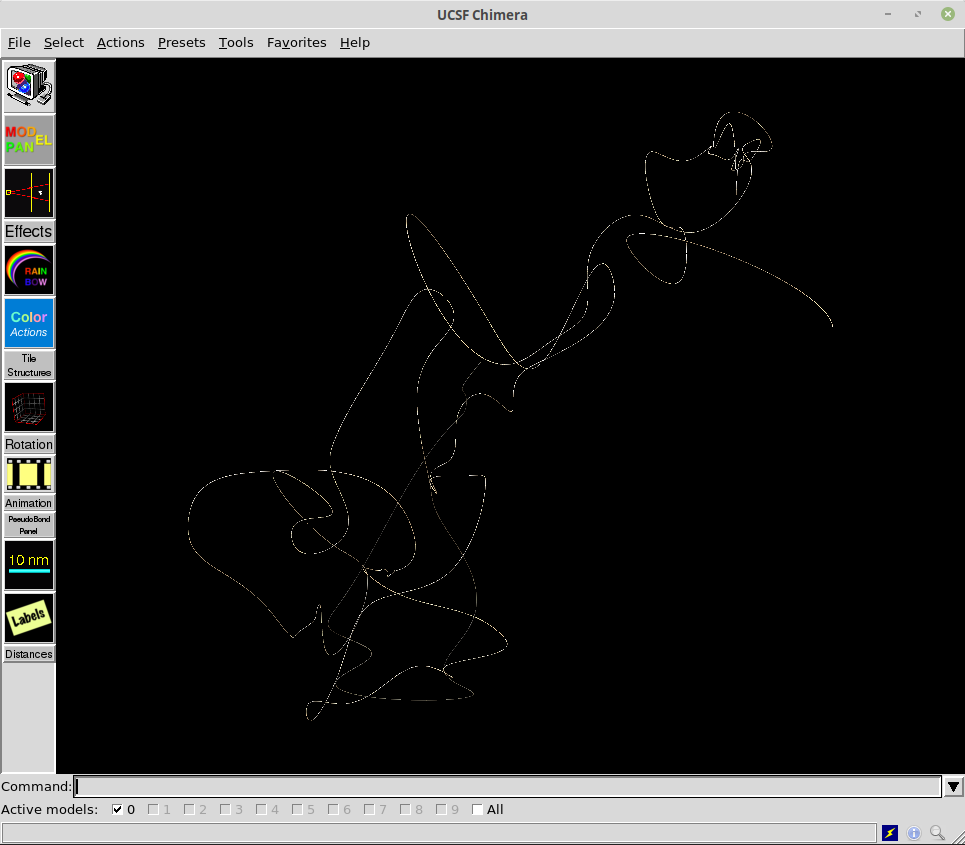

Supplement: Supplementary file 2 — Supplementary Information 2. [file 41598_2022_12568_MOESM2_ESM.zip › ChromoLooping-main/data/readme_images/UCSF Chimera_095.png]

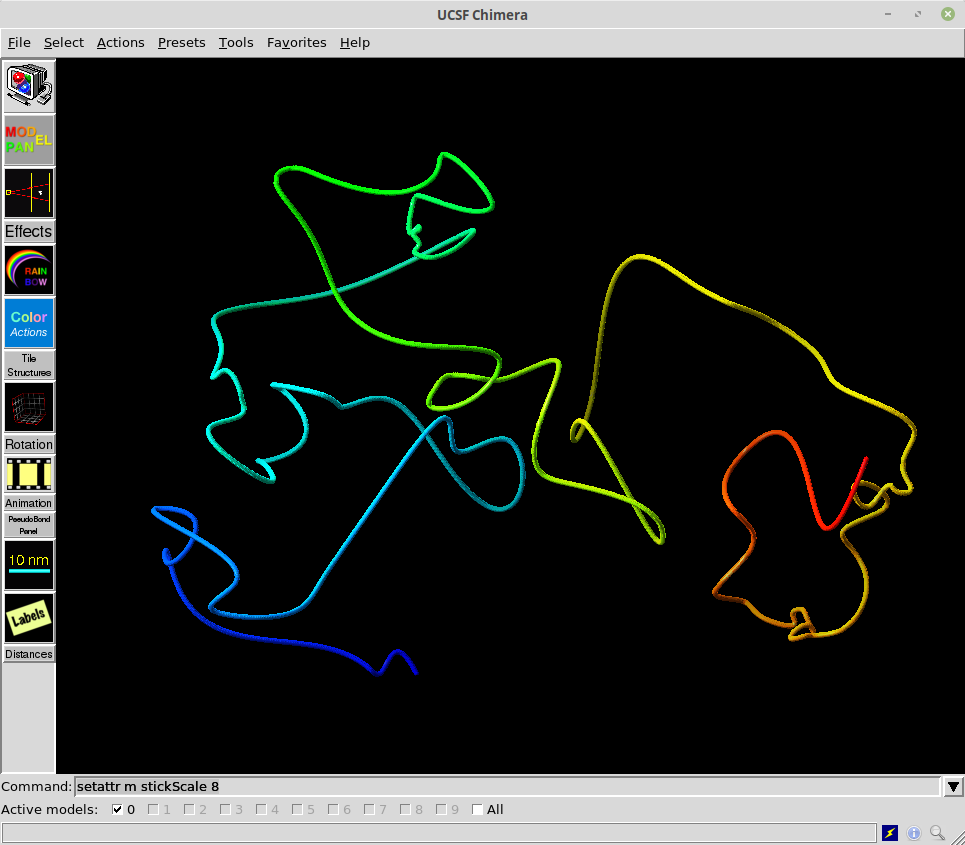

Supplement: Supplementary file 2 — Supplementary Information 2. [file 41598_2022_12568_MOESM2_ESM.zip › ChromoLooping-main/data/readme_images/UCSF Chimera_096.png]

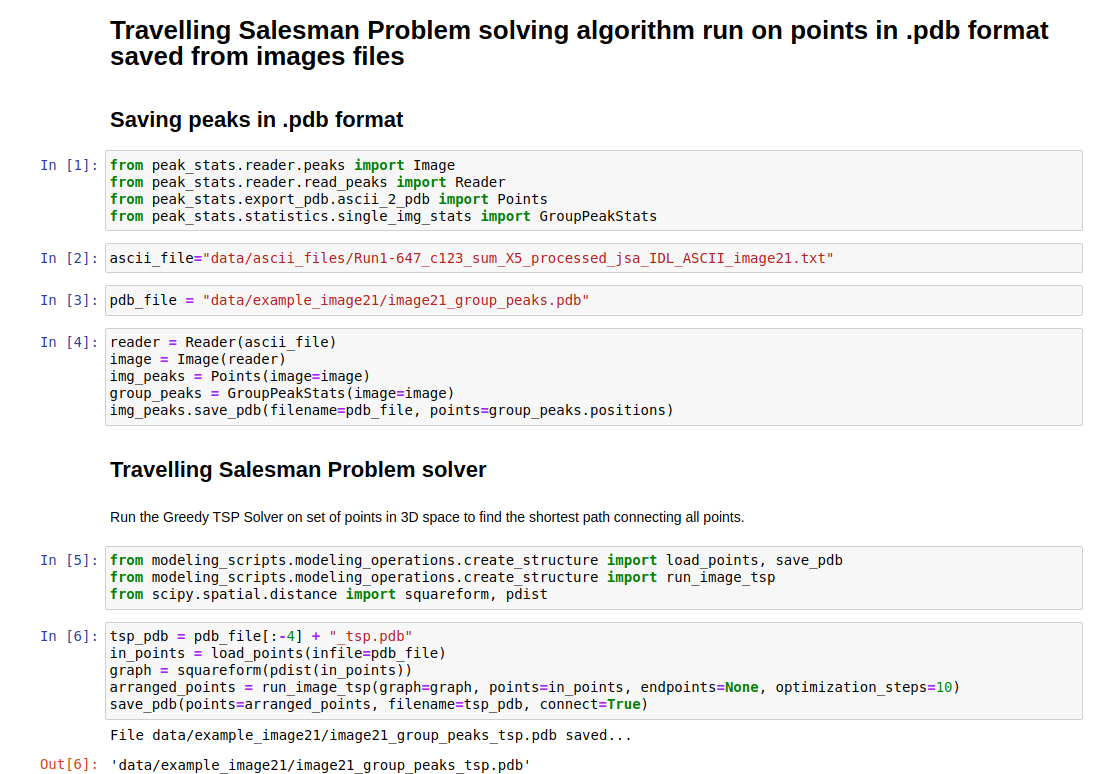

Supplement: Supplementary file 2 — Supplementary Information 2. [file 41598_2022_12568_MOESM2_ESM.zip › ChromoLooping-main/data/readme_images/modelling_notebook_examples.png]

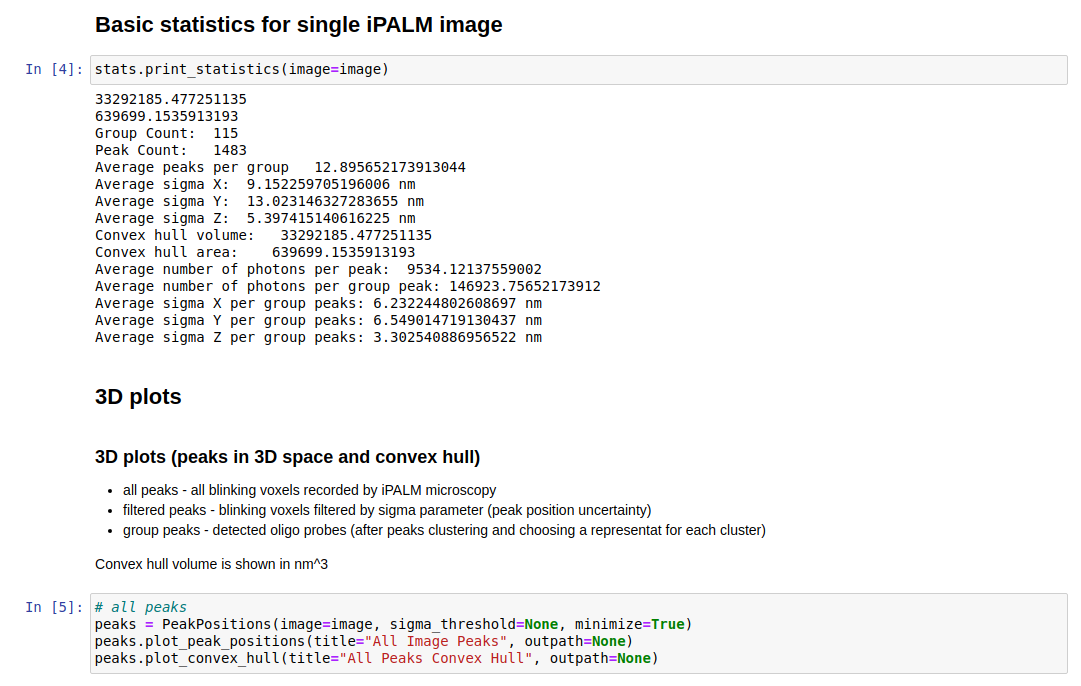

Supplement: Supplementary file 2 — Supplementary Information 2. [file 41598_2022_12568_MOESM2_ESM.zip › ChromoLooping-main/data/readme_images/statistics_example.png]
